# Further Insights on the Migration Biology of Monarch Butterflies, *Danaus plexippus* (Lepidoptera: Nymphalidae) from the Pacific Northwest

**DOI:** 10.3390/insects12020161

**Published:** 2021-02-14

**Authors:** David G. James, Linda Kappen

**Affiliations:** Irrigated Agriculture Research and Extension Center, Department of Entomology, Washington State University, Prosser, WA 99350, USA; humbugkapps@hotmail.com

**Keywords:** overwintering sites, tagging, recoveries, residency, air quality, *Ophryocystis elektroscirrha*

## Abstract

**Simple Summary:**

Monarch butterflies, *Danaus plexippus*, are known the world over for their iconic long-distance migration from the eastern United States and Canada to Mexico for overwintering. In this study, we shed more light on the less well-known migration of monarchs in the Pacific Northwest of North America. Utilizing the efforts of citizen scientists who captive-reared monarchs and tagged them, we confirmed that the majority of monarchs in Washington and Oregon migrate hundreds of kilometers south during late July–October to overwinter at sites on the California coast. However, some eastern Washington and most Idaho monarchs tended to migrate towards the southeast and may have an alternative winter destination, possibly Mexico. Overwintering monarchs in coastal California remain at sites for 2–3 months and can live for up to ten months. A small number of fall migrants may eschew overwintering and join winter-breeding populations in inland central and southern California. Wildfire smoke and infection with a protozoan parasite does not appear to greatly interfere with the survival and migration success of migrating monarch. Our data improve our understanding of western monarch migration, serving as a basis for further studies and providing information for conservation planning.

**Abstract:**

The fall migration of monarch butterflies, *Danaus plexippus* (L.), in the Pacific Northwest was studied during 2017–2019 by tagging 14,040 captive-reared and 450 wild monarchs. One hundred and twenty-two captive-reared monarchs (0.87%) were recovered at distances averaging 899.9 ± 98.6 km for Washington-released and 630.5 ± 19.9 km for Oregon-released monarchs. The greatest straight-line release to recovery distance was 1392.1 km. A mean travel rate of 20.7 ± 2.2 km/day and maximum travel of 46.1 km/day were recorded. Recovery rates were greater for Oregon-released monarchs (0.92%) than Washington-released (0.34%) or Idaho-released monarchs (0.30%). Most monarchs (106/122) were recovered SSW-S-SSE in California, with 82 at 18 coastal overwintering sites. Two migrants from Oregon were recovered just weeks after release ovipositing in Santa Barbara and Palo Alto, CA. Two migrants released in central Washington recovered up to 360.0 km to the SE, and recoveries from Idaho releases to the S and SE suggests that some Pacific Northwest migrants fly to an alternative overwintering destination. Monarchs released in southern Oregon into smoky, poor quality air appeared to be as successful at reaching overwintering sites and apparently lived just as long as monarchs released into non-smoky, good quality air. Migration and lifespan for monarchs infected with the protozoan parasite, *Ophryocystis elektroscirrha* (McLaughlin and Myers), appeared to be similar to the migration and survival of uninfected monarchs, although data are limited. Our data improve our understanding of western monarch migration, serving as a basis for further studies and providing information for conservation planning.

## 1. Introduction

The monarch butterfly, *Danaus plexippus* (L.), is an iconic species in North America and throughout the world, capturing widespread societal interest from children to activists to politicians [1]. In recent times, it has become a flagship species for pollinator conservation in North America, with many groups suffering a substantial worldwide decline in abundance and diversity [2]. Since the mid-1990s, monarch populations in the eastern and western US have suffered declines of ~80–95% [3,4]. The population west of the Rockies is at least three orders of magnitude smaller than the eastern population, and the recent decline has resulted in the smallest populations ever recorded at overwintering sites in coastal California [5]. Until recently, unlike the eastern population, relatively little was known about many aspects of the biology and ecology of western monarchs [6]. However, research during the past five years has provided information on habitats, host plants, breeding [7,8,9,10], and migration [11,12,13,14]. Research has also helped document the continuing decline of western monarch populations [4,5,15].

Pyle [6] suggested that “our understanding of monarch migration in the western population was based more on assumptions, limited observations and intuition rather than on good scientific evidence.” However, recent tagging studies in the southwest [11,14] and Pacific Northwest (PNW) [12,13] have provided important data on the fall migration of monarchs west of the Rocky Mountains. James et al. [13] showed that most recovered monarchs, captive-reared and tagged in Washington and Oregon during late summer and fall 2012–2016, migrated SSE to SSW to coastal California to join overwintering colonies at sites from Bolinas in the north to Carpinteria in the south. In addition, some tagging evidence was also obtained for the S to SE movement of migrating monarchs from eastern Washington and Idaho, which corroborated Pyle’s observations of monarchs migrating to the S and SE in different parts of the PNW [6]. One monarch released in Walla Walla, Washington, was recovered 724 km to the SE in Utah. No Idaho-released monarchs were recovered in California, but two were found ~100 km south of release locations, suggesting they were not heading to California [13].

This study provides additional data on the destinations, rate of travel, survival and lifespan for migrating monarchs from the PNW emanating from citizen scientist captive-rearing and tagging of monarchs during 2017–2019. Captive-rearing of monarchs has received some criticism in recent years with suggestions that it may reduce migration success [16], although our earlier study [13] indicated captive-reared monarchs were successful in reaching overwintering sites and living long lives. In this study, we increase our database of captive-reared, tagged and recovered monarchs from 60 to 182 and asked whether the pattern of migration we saw in our earlier study [13] of predominant south-southwestward movement to California and limited south-eastward movement from Washington and Idaho, was maintained. We also wanted to confirm or refute the low recovery rates of monarchs tagged in Idaho. James [12] reported on a tagged migrant from Oregon found in Santa Barbara, CA ovipositing. In this study, we asked whether we could detect further instances of migrants from the PNW becoming reproductive in CA. We also obtained additional data on longevity, residency and inter-colony movement at coastal overwintering sites in California. Opportunities arose during the study that allowed us to collect limited data on the impacts of wildfire smoke and infection by a protozoan parasite on the success of migration and adult lifespan. The data here, together with earlier information [13], combine to create an improved database from which to base a better understanding of monarch migration from the Pacific Northwest.

## 2. Materials and Methods

Captive monarch rearing was conducted annually during 2017–2019 by WSU personnel and citizen scientists at multiple locations in Washington, Oregon, Idaho, and far northern California (Table 1). In all cases, a single monarch brood, usually from locally derived gravid females, was reared each year by collaborators during July–October.

### 2.1. Washington

Selected inmates at the Washington State Penitentiary (WSP), Walla Walla, reared and tagged monarchs each year. Eggs and first instar larvae produced by captive *D. plexippus* females obtained from breeding habitats in central Washington were provided by DGJ to WSP inmates in late August–early September, who then reared the larvae to adulthood indoors in large cages made from plastic and muslin on cut and/or potted showy milkweed (*Asclepias speciosa* Torr.) at temperatures of 20–30 °C under naturally declining daylengths (windows) [17]. Adults were tagged by inmates and released on the grounds of the Penitentiary or taken to nearby locations for release by Penitentiary staff. Monarch rearing was used by WSP as a program to enhance the mental health of inmates [17]. Captive-rearing and tagging were also conducted each year (August–September) by personnel and citizen scientists at Prosser (WSU staff), Yakima (Cowiche Canyon Conservancy docents and volunteers), and in Spokane and Seattle (members of the Washington Butterfly Association). All captive-reared monarchs were derived from eggs laid by wild females obtained from breeding habitats in central WA, supplied by DGJ. Participants in Yakima (n = 12–20), Spokane (n = 8) and Seattle (n = 3–12) were given tutorials in monarch health, rearing and tagging at the beginning of the project and maintained contact during rearing with WSU via customized monarch rearing Facebook pages. All monarchs reared in Washington were fed on potted or cut *A. speciosa* (Yakima/Spokane) or swamp milkweed (*Asclepias incarnata* L.) (Seattle), exposed to temperatures of 20–30 °C indoors and naturally declining daylengths via windows. Collapsible muslin cages (at least 30 × 30 × 30 cm^3^) were used for rearing. Thirty wild monarchs at a summer breeding site in central Washington were tagged during August–September 2017, but no wild monarchs were tagged in Washington during 2018–2019.

### 2.2. Idaho

Monarchs were captive-reared and tagged each year (20–30 °C), during August–September indoors on potted or cut *A. speciosa* in muslin cages under naturally declining daylengths (windows) in northern Idaho (Bayview, Potlatch) by Bill Harryman and/or Bill Ament (Idaho Dept Fish and Game) and in southern Idaho (Boise) by Dave Hopper (US Fish and Wildlife Service). Monarchs reared in northern Idaho originated from livestock obtained from a mid-California butterfly farm, but those in southern Idaho originated from locally obtained wild females. Nearly 300 wild monarchs were tagged during August–September in breeding areas in southern Idaho in 2017, but less than 30 were tagged during this time period in each of 2018 and 2019.

### 2.3. Oregon

A large number (21–39) of individual citizen scientists in Oregon participated in this program annually, each rearing a varying number (3–600) of monarch butterflies in protected home gardens (~10% of rearers) or indoors during August–October. All captive-reared monarchs were locally sourced from eggs or larvae found within Oregon, often in the back yard or property of the citizen, and were mostly reared in muslin cages on narrow-leaved milkweed (*A. fascicularis* Dcne.) and/or showy milkweed (*A. speciosa*) at 20–30 °C under naturally declining daylengths provided by windows. Those reared outdoors occasionally experienced temperatures >30 °C and <20 °C for short periods. In 2019, a year of very low natural abundance of monarchs in the PNW, 88.9% of monarchs reared and tagged in Oregon originated from eggs laid by a few wild females in Brookings, OR, and transferred to other locations. Limited numbers of wild monarchs (1–59/year) were also tagged in Oregon.

### 2.4. Far Northern California

Three citizen scientists in far northern California (located at Tulelake, Smith River, Montague) reared and tagged 8–103 monarchs indoors in muslin cages on *A. speciosa* or *A. fascicularis* during August–October each year at 20–30 °C under naturally declining daylengths (windows), sourced from local wild eggs and early instar larvae.

### 2.5. Tagging

All monarchs were tagged with a single tag placed on the discal (or “mitten-shaped”) cell on the ventral surface of a hindwing. Tags were obtained from MonarchWatch.org and customized with a serial number and a Washington State University email address (monarch@wsu.edu) for contact. For all tagged monarchs, the date of tagging/release, name of tagger, tag number, location of release and the sex of the butterfly were recorded.

### 2.6. Tag Recoveries

Tagged monarchs were primarily recovered by citizens sighting or photographing butterflies. Most recoveries were made at overwintering sites on the California coast, but a number were made at locations en route to the overwintering sites and occasionally post-overwintering. In Santa Cruz, CA, home to a number of overwintering sites, John Dayton, a monarch biologist, visited three major sites (Natural Bridges State Beach, Lighthouse Field State Beach, Moran Lake Park) weekly during October–March searching for tagged monarchs in each year of the study. In addition, a weeklong visit to overwintering sites from Pismo Beach in the south to Bolinas in the north was made in late November 2017 (by both authors) and 2019 by the senior author to search for tagged monarchs. Virtually all recoveries of tagged monarchs were supported by photographic evidence. Recoveries of monarchs sighted within a few days, and 10 km of the release point were not considered evidence of migration and are not reported.

### 2.7. Impact of Wildfire Smoke and Air Quality on Tagged Monarch Recovery

The impact of wildfire smoke on monarch survival and migration in 2017 and 2018 was opportunistically assessed in selected releases of monarchs from southern Oregon. During August–October 2017, 307 tagged monarchs were released in Brookings, OR, and the air quality index (AQI) was recorded for each release date (https://www.airnow.gov/aqi/aqi-basics/, accessed on 13 February 2021). AQI levels of <100 indicate acceptable air quality, while levels of 100–200 indicate unhealthy air for most people. AQI levels >200 precipitate a health alert, and levels >300 indicate an emergency or hazardous health conditions (https://www.airnow.gov/aqi/aqi-basics/, accessed on 13 February 2021). Of the 307 Brookings-released monarchs, 231 were released into non-smoky “healthy air” (AQI <100) and 76 were released into smoky “unhealthy air” (AQI >100). During August–October 2018, 450 tagged monarchs were released in the Ashland/Medford area of southern OR with the AQI recorded for each release date (http://www.ashland.or.us/SectionIndex.asp?SectionID=534, accessed on 13 February 2021). Of these, 245 monarchs were released into non-smoky “healthy air”, and 205 were released into smoky “unhealthy air”. The incidence of tagged monarchs from these releases recovered in California, along with data on lifespan and distance traveled, was recorded. Where appropriate, a Student’s *t-*test was used to analyze these data.

### 2.8. Impact of a Protozoan Parasite on Tagged Monarch Recovery

*Ophryocystis elektroscirrha* (OE) is a protozoan parasite that naturally infects monarchs [18]. Heavy OE infections are considered detrimental to eastern US monarchs by reducing or impairing longevity, eclosion, mating success, fecundity and flight ability [19,20,21]. In 2017, monarchs in our WSU laboratory developed an increasing incidence of OE during rearing in August–September (this did not occur in other years). Tagged monarchs were assessed for OE prior to release using the tape count method in which sticky tape is placed against the abdomen and then placed on white paper for examination under a stereomicroscope [22]. Infected monarchs had individual loads of 100–1000 spores, but all were apparently fit with no wing deformation or other physical problems and were able to fly strongly when released. The recovery of OE and non-OE infected tagged monarchs in coastal California overwintering colonies was recorded, along with data on longevity and distance traveled.

## 3. Results

**Figure 1:**
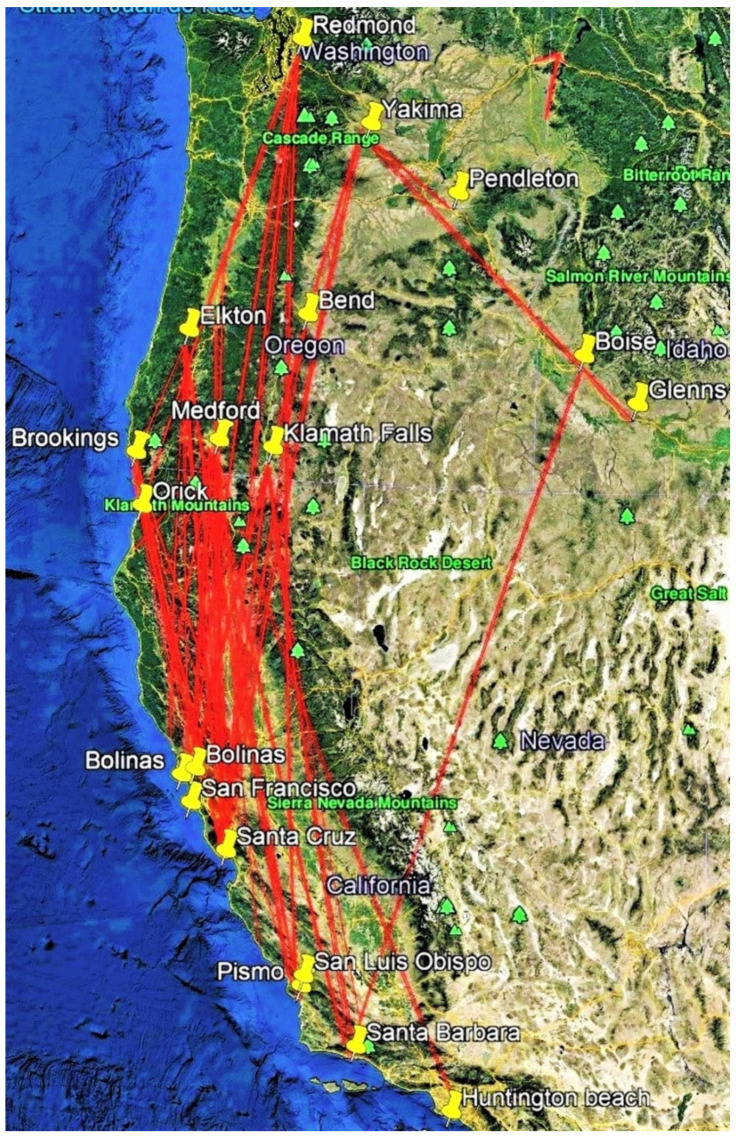
Release–recovery lines (from N to S) for captive-reared tagged monarchs released in the Pacific Northwest during 2017–2019.

**Figure 2:**
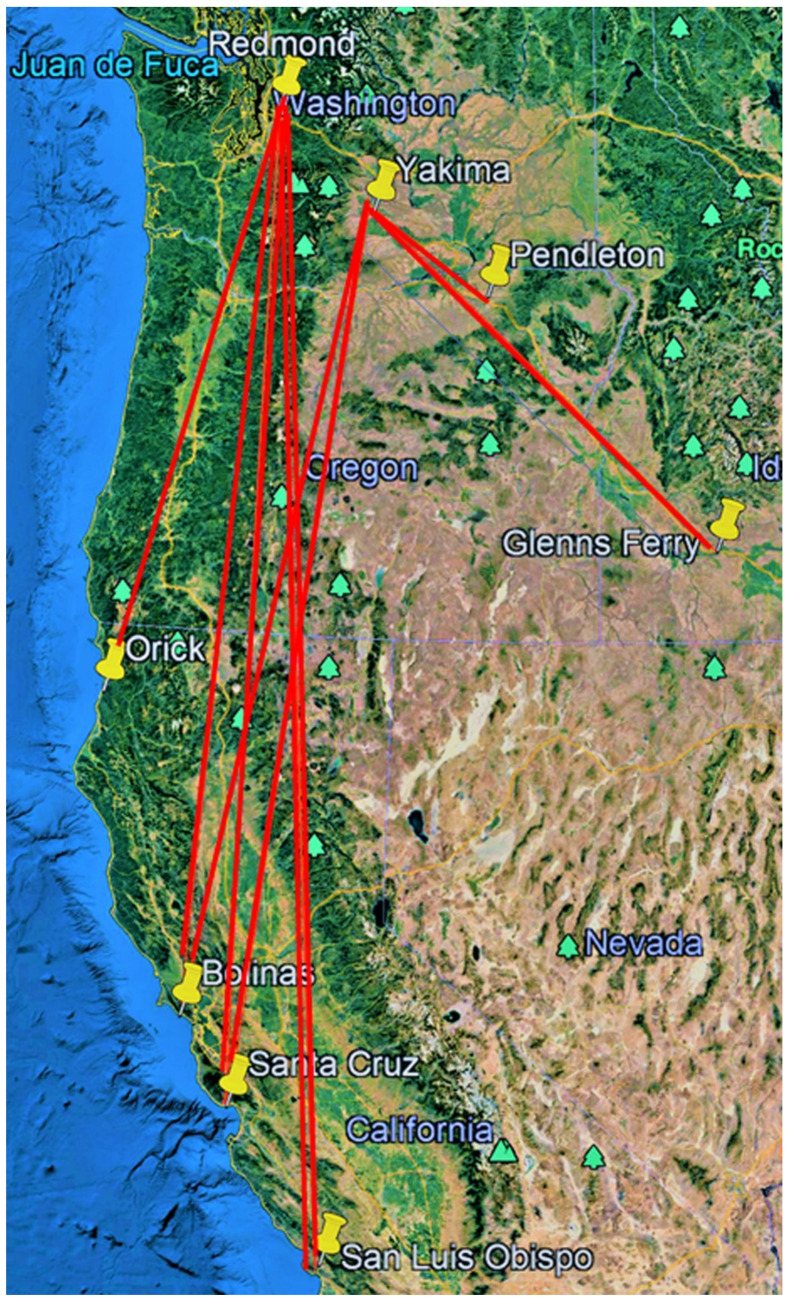
Release–recovery lines (N to S) for captive-reared tagged monarchs released in Washington during 2017–2019.

**Figure 3:**
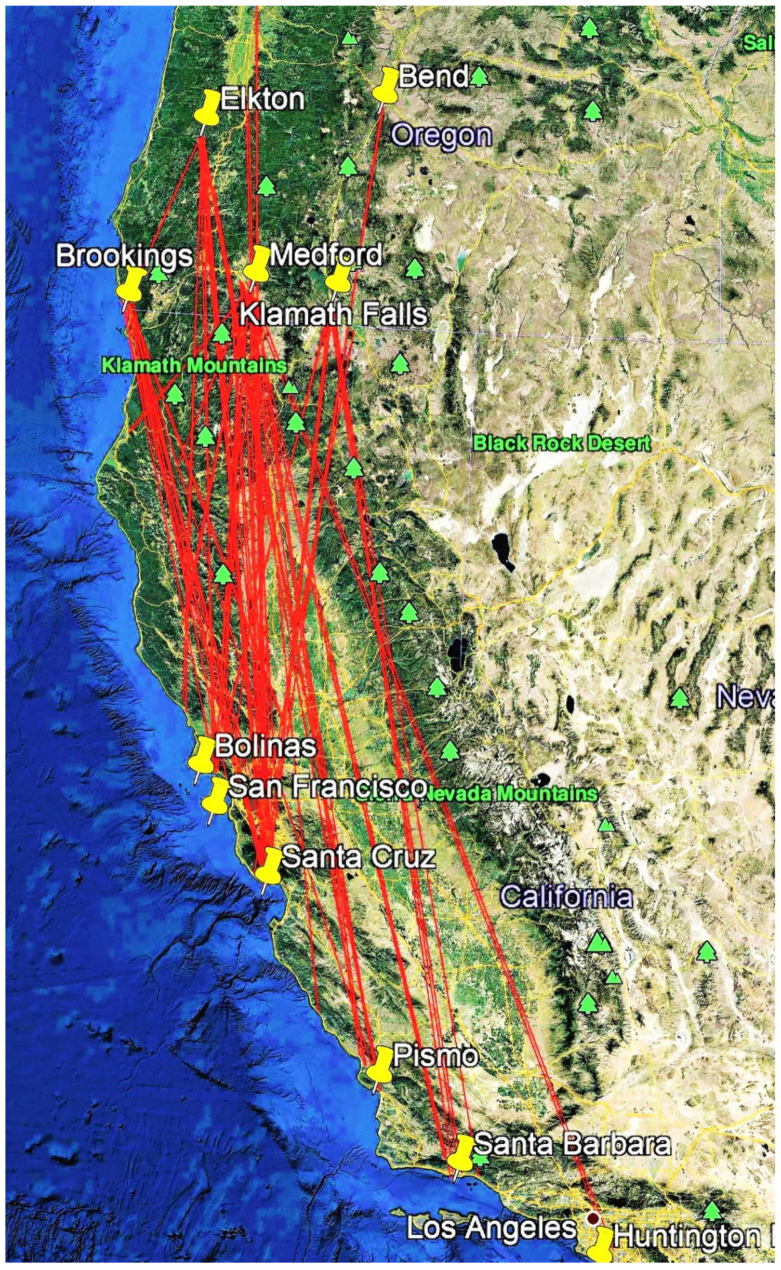
Release–recovery lines (N to S) for captive-reared tagged monarchs released in Oregon during 2017–2019.

**Figure 4:**
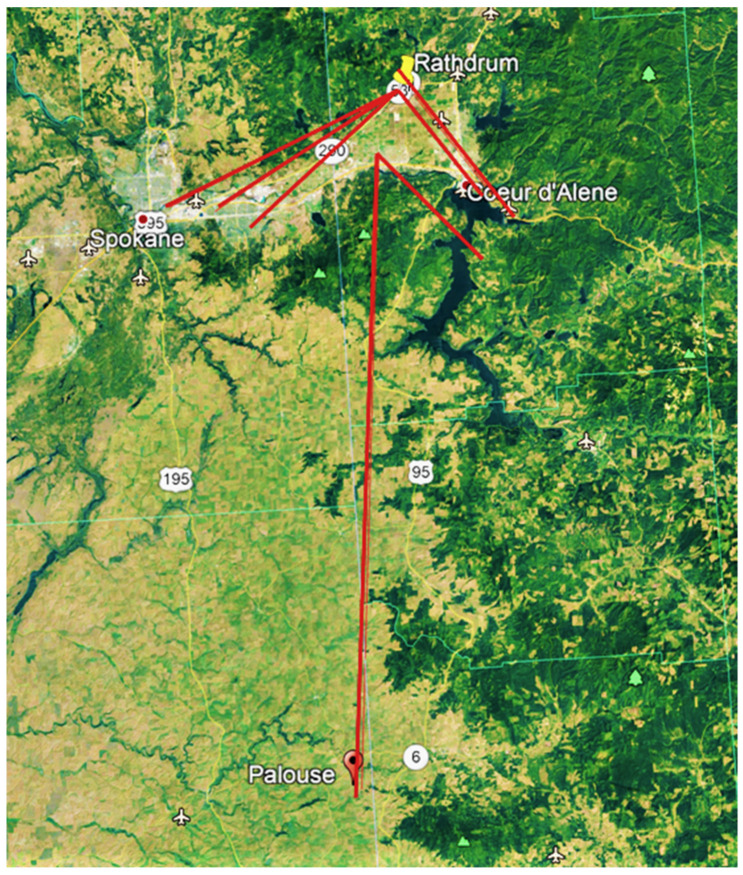
Release–recovery lines (N to S) for captive-reared tagged monarchs released in Idaho during 2017–2019.

Fourteen thousand and forty monarchs were reared and tagged in the PNW during 2017–2019. In addition, 450 wild monarchs were tagged, giving a grand total of 14,490 monarchs tagged over three years (Table 1). One hundred and twenty-two tagged monarchs were recovered, representing 0.87% of the captive-reared butterflies tagged (Table 2, Table 3 and Table 4, Figure 1, Figure 2, Figure 3 and Figure 4). No wild monarchs were recovered. Migration occurred in monarchs released on 30 July and 1 August, while the latest migratory flight occurred in a monarch released from Brookings, OR on October 14. The mean recovery rate over three years was 0.80% (range: 0.49–1.2%), with the greatest recovery rate in 2017 (1.2%). The mean annual recovery rate of Washington-released monarchs was 0.34% (range 0.18–0.57%), lower than for Oregon-released monarchs, 0.92% (range 0.71–1.24%). The mean recovery rate of Idaho-released monarchs was lowest at 0.30% (range 0.00–0.79%), and apart from one, all were found in Idaho. Most recovered monarchs (106/122, 86.9%) were found in California, SSW-S-SSE of release points, and most of these (82, 65.6%) were at coastal overwintering sites from Bolinas in the north to Carpinteria in the south (Table 2, Table 3 and Table 4, Figure 1).

**Figure 5:**
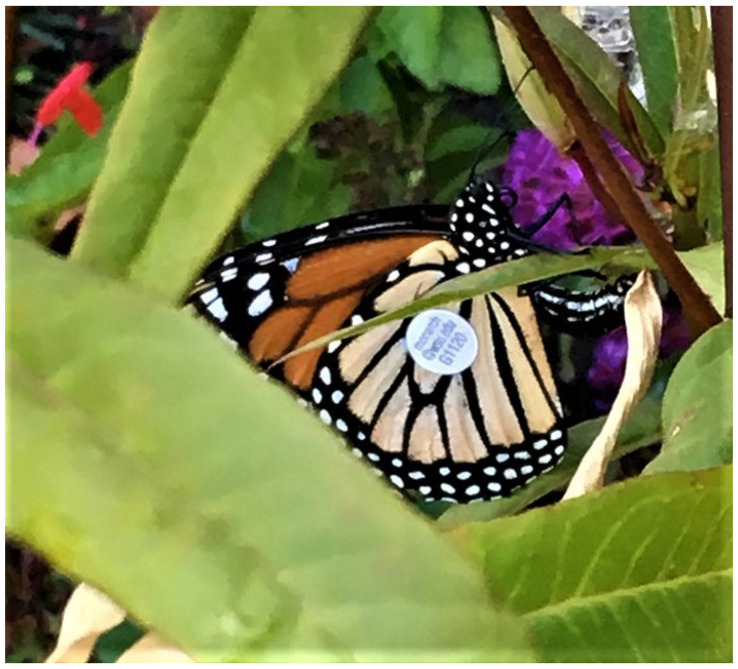
G1120, a female monarch reared and released in Talent, Oregon on 14 September 2019 by Belinda Vos and photographed 40 days later on 24 October 2019 ovipositing on backyard milkweed in Palo Alto, California, after migrating 537.5 km. Photo by Karen Krimmer Easton.

Two recoveries were made of tagged females that had migrated 877.0 and 537.5 km from Oregon and were seen ovipositing on tropical milkweed (*Asclepias curassavica* L.) in suburban backyards in Santa Barbara (# B6679, Table 2, [12]) and Palo Alto, CA (# G1120, Table 4, Figure 5). Two individuals released in Yakima, WA were recovered 173.8 and 360.0 km to the SE in Pendleton, OR and Glenns Ferry, ID, respectively (Figure 2). Four of the eight recoveries of monarchs released in Idaho were recovered at locations 16.1–96.6 km S-SE of release points (Figure 4). Only one individual (0.04%) released in Idaho (from 2458 tagged) was recovered in California (Santa Barbara, 1065.4 km SSW, Figure 1). Of the 122 recovered monarchs, 89 (73.0%) originated in Oregon (45.7% of PNW-tagged butterflies), 22 (18.0%) originated in Washington (35.8% of PNW-tagged butterflies), 8 (6.6%) originated in Idaho (17% of PNW-tagged butterflies) and 3 (2.4%) originated in far northern California (1.5% of PNW-tagged butterflies) (Figure 1, Figure 2, Figure 3 and Figure 4).

### 3.1. Recoveries of Tagged Monarchs at Overwintering Sites

Eighty-two Washington and Oregon-released monarchs were found at 18 coastal California overwintering sites during October–March (Table 2, Table 3 and Table 4). Fifty-three (64.6%) of these were found at the three major overwintering sites (Natural Bridges State Beach, Lighthouse Field State Beach, Moran Lake Park) in Santa Cruz. The second most popular location for recoveries was Bolinas (11 at two overwintering sites), and six were found at the Pismo State Beach overwintering site. Single individuals were found at twelve other overwintering sites.

### 3.2. Residency and Inter-Colony Movement of Tagged Monarchs at Overwintering Sites

Forty-seven tagged monarchs sighted at overwintering sites were re-sighted two to twelve times in the same colonies 6 to 139 days later (Table 5, Table 6 and Table 7). Fifteen tagged monarchs were seen in two different overwintering colonies, and two (B5593, B6578) were seen in three Santa Cruz overwintering colonies. Tagged monarchs resided (as judged by re-sightings) at overwintering sites for up to 139 days. However, mean residency in each year varied from one to two months (mean = 60.6 ± 6.0 days, n = 47) (Table 5, Table 6 and Table 7).

### 3.3. Rate of Travel of Tagged Monarchs

Thirty-one tagged monarchs were recovered during the fall migration either at locations en route or as early colonizers at overwintering sites. Mean travel rates ranged from 18.7 ± 3.8 km/day in 2017 to 23.3 ± 1.9 km/day in 2019 with an overall rate of 20.7 ± 2.2 km/day. Maximum travel rates of 41.4 and 46.1 km/day occurred in 2018 and 2017, respectively (Table 8).

### 3.4. Distance Traveled by Tagged Monarchs

The greatest straight-line release point to recovery point distance recorded was 1392.1 km from Redmond, WA to Avila Beach, CA (Table 2). The greatest distance traveled by an Oregon-released monarch was 1042.9 km from Talent to Huntington Beach, CA (Table 4). The greatest distance traveled by an Idaho-released monarch was 1065.4 km from Boise to Santa Barbara, CA (Table 2). The mean distance traveled by Washington-released monarchs was 899.9 ± 98.6 km, compared to 630.5 ± 19.9 km for Oregon-released monarchs and 160.2 ± 129.7 km for Idaho-released monarchs.

### 3.5. Length of Life of Tagged Monarchs

The lifespan of tagged monarchs recorded as the interval between tagging and final sighting ranged from 5–312 days (mean = 82.0 ± 5.2 days, n = 122). Removing migrants (i.e., monarchs only seen prior to arrival at overwintering sites) from this analysis increases the mean lifespan to 107.4 ± 4.9 days (n = 88) or almost 3.5 months (Table 2, Table 3 and Table 4). This analysis does not take account of post-overwintering survival. The maximum lifespan recorded in this study (312 days/44.6 weeks/10 months for tag # E5182 in 2018) may be the longest recorded for a western monarch. This individual (not seen at an overwintering site) was captive-reared and released into smoky conditions (see below) at Ashland, Oregon on 18 August 2018, and recovered at Cayucos, California, on 26 June 2019.

### 3.6. Impact of Wildfire Smoke and Air Quality on Tagged Monarch Recovery

Nine (2.9%) captive-reared tagged monarchs from the Brookings, Oregon cohort of 307 released into smoky or non-smoky atmospheric conditions during August–October 2017 were recovered in California (Table 9). Four out of 76 (5.3%) released into smoky (unhealthy-hazardous) air (AQI: 198–304) were recovered, with three alive at overwintering sites 31 to 164 days after release (mean 109 ± 32.5 days) after migrating 455.4 to 618 km (mean 566.4 ± 37.4 km). One individual was recovered after 31 days at Vacaville, CA (455.4 km from Brookings), possibly en route to an overwintering site. Five out of 231 (2.2%) released into non-smoky, healthy air (AQI: < 50) were alive at overwintering sites 48 to 104 days post-release (mean 83.8 ± 11.3 days) after traveling 487.6 to 638.9 km (mean 570.0 ± 28.2 km) (Table 9). Post-release lifespan (*t* = 0.805, 7 df, *p* = 0.447) and migration distance (*t* = −0.0790, 7 df, *p* = 0.939) were not significantly different between the smoky and non-smoky air release cohorts (Student’s *t*-test).

Nine (4.4%) of 205 captive-reared tagged monarchs from the Ashland-Medford cohort of 450 released into smoky, (unhealthy-hazardous) atmospheric conditions (AQI: 100–177) during August–September 2018 were recovered in California after migrating 188.3 to 772.5 km (mean 537.3 ± 60.1 km). Post-release lifespan ranged from 13 to 312 days (mean 88.6 ± 31.2 days) (Table 10). Three of the recoveries were made 13–16 days after release (presumably migrating), and one individual was recovered 312 days after release, presumably following overwintering (Table 10). The remainder were recovered at overwintering sites. No recoveries were made of 245 monarchs released into healthy air (AQI < 100) during the same period.

### 3.7. Impact of a Protozoan Parasite on Monarch Migration and Length of Life

Captive-reared tagged monarchs released from Yakima, WA in 2017, infected or uninfected with OE, were recovered in California at overwintering sites at an apparently similar rate (OE-infected: 6/1473 (0.41%), Non-infected: 2/450 (0.44%). OE-infected monarchs migrated 983.1 to 1084.7 km (mean: 1051.2 ± 20.7 km) with a lifespan of between 57 and 170 days (mean: 116.8 ± 16.2 days) when last seen. Non-infected monarchs migrated 1084.7 km and had lived from 61–169 days (mean: 115.0 ± 54.0 days) when last seen (Table 11).

## 4. Discussion

This study provides additional and supportive data for our earlier study on monarch migration from the PNW [13]. That study conducted during 2012–2016 provided a preliminary understanding of monarch migration in the PNW based on 60 recoveries of tagged monarchs. The current study, conducted during 2017–2019, extends that research, adding a further 122 recoveries of captive-reared tagged monarchs. Our new data broadly support the earlier findings that the majority of monarchs in Washington and Oregon migrate south in the fall (August–October) to overwinter at sites along the California coast. We also provide additional evidence for some south-to-south-easterly movement of tagged monarchs in eastern Washington and Idaho first suggested by Pyle [6] and documented in James et al. [13], suggesting the possibility of an alternative overwintering destination for some PNW populations. While the majority of migrants from the PNW persist as non-reproductive individuals in overwintering colonies, we document two instances of tagged female monarchs migrating to California from Oregon and becoming reproductive after a few weeks, a phenomenon initially noted by James [12]. The fall migration in the PNW begins in late July. The earliest confirmed fall migrant in this study was released on 30 July 2017 in Eugene, Oregon (# B5260). A captive-reared tagged monarch released in Spokane, WA on 20 July 2020 was also apparently migratory, flying 98.2 km SSE in 8 days (James, unpubl. obs.). This study also provides opportunistic but limited data on the lack of impact of wildfire smoke (producing unhealthy atmospheric conditions) on survival, migration success and lifespan of monarchs exposed to these conditions early in adult life. Limited data were also obtained on apparent migration success and lifespan of monarchs infected with the protozoan parasite, *O. elektroscirrha* (OE).

The western US monarch population has always been substantially smaller than that of the eastern US [23,24]. The sparsity of northwestern monarch populations means that it is not feasible to tag enough wild butterflies to provide robust recovery data needed for conclusions about migration. Consequently, this and our earlier study [13] depended on captive-rearing by citizen scientists to generate monarchs for tagging. During the course of this research (2012–2019), 27,820 monarchs were captive-reared and tagged, along with 1323 wild-caught monarchs that were also tagged. One hundred and eighty-two monarchs were recovered (0.62%), and all except one were captive-reared monarchs. The single tagged wild monarch was mistakenly assigned as reared in our earlier study (Tag # A3264, Table 5 in James et al. [13]). During the current study, 122 (0.84%) tagged monarchs (all captive-reared) were recovered from 14,490 released.

The viability of captive-reared monarchs as successful migrants in eastern North America has recently been questioned [25,26,27]. Two of these studies [25,27] depended on flight simulators to conclude that captive-reared monarchs did not show proper orientation southward. However, captive-reared monarchs released in the wild and tracked by radio-telemetry apparently do show correct orientation [28]. Davis et al. [26], working with eastern US butterflies, showed captive-reared monarchs underperformed in three of four physical traits considered important for migration when compared to wild monarchs. Earlier, Steffy [29] showed that captive-reared eastern US monarchs have lower tag recovery rates (0.06%) than their wild counterparts (0.50%). This study and James et al. [13] suggest that captive-reared western US monarchs have little difficulty migrating to overwintering sites and living extended lives (up to 10.5 months). Although our number of wild-tagged monarchs (1323) was substantially lower than captive-reared, tagged monarchs (27,820), our 0.62% recovery rate for captive-reared monarchs predicts that at least eight wild-tagged monarchs should be recovered. Instead, we had a single wild tag recovery (0.08%) in eight years. It is possible that western and eastern monarchs differ in their response to captive rearing. A recent study [30] showed that despite no genetic difference, eastern monarchs are capable of superior flight performance in the laboratory compared to western monarchs. It is also possible that the more modest migration of western compared to eastern monarchs may serve to mask any negative influences of captiverearing if they exist.

The discovery of two tagged female monarchs (# B6679, # G1120) that had migrated 537–877 km from Oregon to California and become reproductive indicates that not all migrants from the PNW contribute to the high profile non-reproductive coastal overwintering populations in California. Monarch # B6679 was found ovipositing in a Santa Barbara backyard, joining the year-round breeding population of monarchs present in parts of southern California [12,31]. Monarch # G1220 was found ovipositing on backyard milkweed in Palo Alto, about 45 km south of San Francisco, where breeding populations of monarchs are not usual during winter but have become more frequent in recent years (James, unpubl. data). The frequency of assimilation of PNW fall migrants into winter-breeding California populations of monarchs is unknown but could be significant and increasing, especially in years of above-average fall temperatures. If the number of migrants that become reproductive is significant, then this may be an additional factor contributing to the recently documented contemporary smaller and declining populations at overwintering sites [4]. However, some research has shown that non-reproductive status may not be a reliable indicator of migratory status [32]. Research is urgently needed on this possibility of migrants from the PNW forming winter breeding populations in the San Francisco area. Observations reported online on social media and natural history reporting sites during winter 2020/21 suggested a substantial increase in monarch winter breeding activity in the San Francisco area (James, unpubl. data).

Tag recovery rates were greatest for Oregon-released monarchs (0.92%) and lowest for Idaho-released monarchs (0.30%), with Washington-released monarchs intermediate (0.34%). These recovery rates are similar to those reported for 2012–2016 [13]. They are also within the range reported for Arizona [11] and eastern US monarchs [33]. However, it is likely that the recovery rate for eastern US monarchs is an underestimate because it only uses individuals found dead at the Mexico overwintering sites [33]. If live tagged butterflies were counted in Mexico, the recovery rate would likely be much higher. In years with weather-related mass mortality events in Mexico, tag recovery rates reach 3–4% [33]. If tag recovery rates of ~1% are a true reflection of migration success in western monarchs, this could mean that migration mortality is greater in the west than in the eastern US, despite the much shorter travel distances. Continued tagging of the PNW population is needed to strengthen the conclusions drawn from our data to ensure we are not overlooking other possible outcomes.

While the majority of recoveries occurred in California, usually at overwintering sites, two monarchs released at Yakima in eastern WA were found 173.8 and 360.0 km to the SE. These individuals appeared not to be heading towards the California coast, similar to one individual released from Walla Walla, WA in 2012 and found at Brigham City, UT, (724.0 km SE) [13]. Similarly, half of the recoveries of Idaho-released monarchs in the current study were found S-SE of release points. Two of three Idaho-released monarchs reported in James et al. [13] were found to the S and SE. More recently, a tagged monarch released in Spokane, WA, was found a few days later 97.5 km to the SSE in Moscow, Idaho (James, unpubl. data). Together, these data support the suggestions of Pyle [6] and James et al. [13] that some monarchs in eastern WA and ID orient S and SE during the fall migration. The overwintering destination of S and SE orienting monarchs is uncertain but may include Arizona and/or Mexico. The lower recovery rates of tagged monarchs released in ID and eastern WA may be a consequence of a longer and more hazardous migration over sparsely vegetated and populated regions of ID, UT and AZ. Although 3655 monarchs were tagged in ID during 2012–2019, yielding 11 recoveries (0.30%), it is clear that many more need to be tagged in this state to provide data on destinations.

The mean lifespan (from release to final sighting) of monarchs at overwintering sites in this study was comparable (107.4 days) to that reported in James et al. [13] (98.9 days). However, the longest lifespan for a tagged monarch in the current study (312 days or just over 10 months, # E5182) was nearly twice that recorded in James et al. [13] (164 days), and it appears to be the longest-lived monarch reported from the western US to date. Data on release–recovery intervals showed distances of up to 46.1 km a day can be traveled by migrants, with a mean of 20.7 km traveled per day. James et al. [13] obtained a higher daily travel mean rate of 35.1 km with a maximum of 62.6 km/day. Combining the data from the two studies (n = 44) provides a mean daily rate of 25.0 km/day, lower than the 45.0 km/day reported by Brower et al. [34] for migrating monarchs in the eastern US. Eastern US monarchs were shown to have superior flight performance than western monarchs [30], and most have a substantially longer distance (up to 4000 km) to travel to reach the overwintering area in Mexico. Monarchs in this study migrated from the PNW for distances of 465.1–1392.1 km to reach overwintering sites in California, similar to the shortest (486.0 km) and longest (1360.0 km) distances reported by James et al. [13]. Since the commencement of tagging in 2012, only one (0.03%) of 3655 monarchs tagged in Idaho was recovered in California. This is an extremely low recovery rate and further supports the hypothesis that a good proportion of Idaho monarchs migrate in a southerly and/or south-easterly direction, possibly heading for overwintering sites in Arizona or Mexico.

Sixty-seven percent of tag recoveries occurred at 18 overwintering sites in coastal California. If all data obtained since 2012 are considered, 72% of tag recoveries occurred at 24 overwintering sites. Given the discrete nature of most overwintering colonies that occupy a small area and the relatively low numbers, we believe that we detected most of the tagged monarchs present. This was particularly true for sites like the Santa Cruz sites that were visited weekly or fortnightly and for public sites with many human observers like Pismo beach. However, at sites with large populations that we only visited once or twice (e.g., Bolinas), we may not have sighted all of the tagged butterflies present. Similar to James et al. [13], a majority of recovered monarchs (64.6%) in this study were recorded at overwintering sites in Santa Cruz. While clearly a favored location with three major sites, the preponderance of recoveries in Santa Cruz was no doubt strongly influenced by the weekly inspection of overwintering colonies in this location during October–March. These weekly inspections also facilitated the collection of data on butterfly residency at overwintering sites in Santa Cruz by individual tagged monarchs. Residency at single or multiple overwintering sites of up to 131 or 139 days, respectively, was recorded, similar to the 123 days at single sites recorded by James et al. [13]. James et al. [13] reported two tagged monarchs were found at two different Santa Cruz overwintering sites at different times during the winter. In this study, fifteen monarchs were reported from two or three overwintering sites in Santa Cruz, suggesting that movement between nearby colonies can be common. The three major overwintering sites in Santa Cruz are separated by 2.85–7.47 km.

A potential hazard to migrating monarchs in the western US is the incidence of wildfires and associated profuse smoke and poor air quality that has increased since the turn of the century [35]. No information exists on the possible impact of wildfire smoke on monarch migration. Hegedus et al. [36] suggested that by interfering with sky polarization, forest fire smoke might be responsible for the reported disorientation of some migratory insects in forest fire season in British Columbia [37]. Wildfires also produce emissions of gases like CO_2_; that might affect the orientation, flight and survival of invertebrates. The limited data collected in this study for tagged monarchs released into smoky, poor air quality conditions, compared to individuals released into good air quality at the same location in the same migration season, suggests little impact on migration and survival. Monarchs released into smoky air were just as successful in reaching overwintering sites and had a similar survival rate as butterflies released into non-smoky air. When recoveries are examined as a percentage of individuals released into either smoky or non-smoky conditions, the recovery rate was higher in smoky (4.4–5.3%) than non-smoky conditions (0–2.2%). The tagged monarch (# E5182) reported in this study as the longest-lived monarch documented in the western US (>10 months) was released into dense wildfire smoke and unhealthy air. However, the data we collected were opportunistic, not part of a planned experiment and are therefore limited. Additional studies are needed before firm conclusions can be made about the impact of wildfire smoke on monarch migration and survival.

Monarchs are commonly infected by the naturally occurring protozoan parasite, *O. elektroscirrha* (OE), and many studies have shown that badly infected adults are smaller, have lower eclosion success, and have reduced longevity and flight performance [19,20]. Infected monarchs are also reported to have reduced fitness for flight and migration [22,38,39]. Although opportunistic and very limited, the data collected on migration success and survival of heavily OE-infected monarchs in this study suggests that OE has little impact on the viability of migrating western monarchs. Both infected and non-infected migrants were recovered in California at an apparently similar rate, migrated similar distances and had a similar lifespan at final sighting. Although our data are limited, they suggest that the shorter distances flown by migrants in the west mean that western monarchs may be less impacted by OE infection than their eastern counterparts. Altizer et al. [22] showed that approximately 30% of the western US monarch population was heavily infected with OE but that this did not affect overwintering mortality. More research on this important issue is needed.

Our data here support the concept previously espoused by James et al. [13] that most PNW monarchs, particularly those from Oregon, fly to central California coastal overwintering sites during the fall. However, it is also clear that some individuals from Washington and Idaho orient S-SE and may overwinter elsewhere, as suggested by Pyle [6] and James et al. [13]. Increased tagging efforts in eastern Washington and Idaho should ultimately reveal the destinations of S-SE orienting migrants from these areas. Some PNW migrants may not join overwintering populations and may instead join winter-breeding populations in California. Recent field observations and reports from California suggests that this phenomenon may be increasing in incidence and importance. The recent widespread availability and planting of the introduced ornamental milkweeds, *A. currassavica* L., *Gomphocarous physocarpus* E. May and *Gomphocarpus fruticosus* (L.) W.T. Aiton, in the San Francisco and Los Angeles urban areas may help divert migrating monarchs towards reproduction as suggested by Satterfield et al. [40]. However, in Australia, winter reproductive and non-reproductive monarchs can exist in the same habitat together, suggesting termination of reproductive dormancy and migration in fall monarchs is dependent on more than host availability [41]. More research is needed to determine the cues involved and the importance of fall migrants to California winter breeding populations in the overall population dynamics and ecology of monarchs in the western US.

The fall migration of monarchs from the PNW is a period of major vulnerability. Factors such as lack of nectar, wildfires, predation, vehicle traffic, pesticides and pollution, frequently occur at high or heightened levels during August–September. Mortality during fall migration is likely to be a significant factor in the population dynamics of monarchs in the western US and may have contributed to recent declines in the west and east [4,42,43,44]. Recent work showed a correlation between the greenness of Texas and migration success [33]. Our research indicates that the bulk of migrating monarchs from the PNW travel through inland areas of Washington, Oregon and northern California. The availability of nectar in this region during August–October is likely to be one of the most important factors affecting monarch migration. Much of the region monarchs fly through is arid, sparsely populated and lacking in late summer and fall nectar resources. However, there are some key arid region nectar sources for monarchs like rabbitbrush (*Ericameria* spp.) available during this period that are likely critical for monarch survival. The majority of tagged migrants observed during this study were seen nectaring on garden ornamentals in townships in arid landscapes, suggesting that country towns may provide “rest stops” for migrating monarchs. Stocking gardens in country towns with fall-flowering plants like asters and buddleja in eastern parts of WA, OR and northern CA should be beneficial for migrants. Although the flight behavior of migrating western monarchs has not been studied, it is likely that they fly at high altitudes during the day like eastern monarchs [45] descending late in the day to “green” areas where they can find trees for roosting. Thus, it is possible that they “choose” towns and river courses for nectaring and roosting because, in arid landscapes, this is where most of the trees are. Ensuring nectar is available at these locations during fall is a simple conservation strategy that has the potential to benefit western monarch populations. Refraining from using pesticides, particularly neonicotinoids [46], in the home garden and public spaces during the migration season will also benefit monarch survival.

## Figures and Tables

**Table 1:** Numbers of monarchs, captive-reared and tagged and *wild-tagged* by organizations and citizen scientists during 2017–2019 (WSP = Washington State Penitentiary, WSU = Washington State University, CCC = Cowiche Canyon Conservancy, WBA = Washington Butterfly Association, IDFG = Idaho Fish and Game, USFWS = United States Fish and Wildlife Service. Italics separate *wild-tagged* from captive.

Location	2017	2018	2019	2017–2019
**Washington**				
WSP (Walla Walla)	494	580	105	1179
WSU (Yakima)	1972	452	121	2545
CCC (Yakima)	192	245	160	597
WBA (Redmond, Spokane)	442	390	0	832
*Wild-tagged*	*30*	*0*	*0*	*30*
Total (WA)	3130	1667	386	5183
**Idaho**				
IDFG (Bayview)	863	875	311	2049
USFWS/IDFG (Boise)	19	0	57	76
*Wild-tagged*	*278*	*28*	*27*	*333*
Total (ID)	1160	903	395	2458
**Oregon**				
Central and Northern	176	68	116	360
Southern	1987	1041	3157	6185
*Wild-tagged*	*59*	*15*	*1*	*75*
Total (OR)	2222	1124	3274	6620
**Far N California**				
Tulelake, Montague, Smith R.	8	31	165	204
*Wild-tagged*	*12*	*0*	*0*	*12*
Total (CA)	20	31	165	216
**Montana**				
Missoula	0	0	13	13
Total (MT)	0	0	13	13
Total (PNW): *Wild*	*379*	*43*	*28*	*450*
Total (PNW): Reared	6153	3682	4205	14,040
Total (PNW): All	6532	3725	4233	14,490

**Table 2:** Release, recovery, distance, direction and lifespan data for monarchs, captive-reared, tagged and released in the PNW during 2017. **OS** = overwintering site. Santa Cruz overwintering sites: LF = Lighthouse Field State Beach, NB = Natural Bridges State Beach, ML = Moran Lake.

Tag #	Sex	Release Location	Date and Tagger	Recovery Location & Date	Length of Life (Days)	Distance (km) and Direction	Finder
**B5629**	F	Gold Hill, OR	Aug 18, *Lee Finney*	Jacksonville, OR Aug 19	1	20.9 SE	*Angela Street*
**B6917**	F	Rathdrum, ID	Aug 18, *Bill Ament*	Coeur d’ Alene, ID Aug 21	3	16.1 SE	*Debbie Rochon*
**C2564**	M	Rathdrum, ID	Aug 20, *Bill Ament*	Spokane, WA Aug 24	4	24.1 SW	*Nicole Carson*
**B6839**	M	Rathdrum, ID	Aug 25, *Bill Harryman*	Spokane, WA Aug 28	3	27.4 SW	*Michelle Fab*
**C4433**	F	Rathdrum, ID	Aug 28, *Bill Harryman*	Spokane, WA Aug 30	2	25.7 SW	*Susan Stephens*
**B6865**	M	Rathdrum, ID	Aug 28, *Bill Harryman*	Coeur d’ Alene, ID Sep 2	4	16.1 SSE	*Paul Klawitter*
**C3416**	M	Redmond, WA	Sep 2, *Connie Granberg*	Redmond, WA Sep 5	3	10.0 SE	*Mike Hamilton*
**C3462**	F	Redmond, WA	Sep 3, *Connie Granberg*	Redmond, WA Sep 5	2	10.0 SE	*Wanda Boot*
**C2878**	M	Post Falls, ID	Aug 22, *Bill Ament*	Coeur d’ Alene, ID Sep 5	14	10.0 SE	*Charlie Miller*
**C4772**	F	Redmond, WA	Sep 3, *Connie Granberg*	Mercer Island, WA Sep 10	7	16.1 SE	*Cindy Lee*
**B5553**	F	Elkton, OR	Sep 6, *B. Slott/K. Hendricks*	Grants Pass, OR Sep 11	5	138.4 S	*Janet Gogue*
**B5554**	F	Elkton, OR	Sep 6, *B. Slott/K. Hendricks*	Brookings, OR Sep 11	5	191.5 SSW	*Paul La Barbera*
**B5518**	M	Elkton, OR	Sep 3, *B. Slott/K. Hendricks*	Jenner, CA Sep 17	14	585.8 S	*Nick Lasater*
**B6679**	F	Klamath Falls, OR	Sep 3, *Akimi King*	Santa Barbara, CA Sep 22	19	877.1 SSE	*Cathy Fletcher*
**B3729**	M	Brookings, OR	Aug 24, *Dennis Triglia*	Vacaville, CA Sept 24	31	455.4 SSE	*“charmedlady”*
**C1143**	M	Yakima, WA	Sep 8, *David James*	Toppenish, WA Sept 21	13	24.1 SSE	*B. and D. Cunningham*
**C4378**	F	Talent, OR	Sep 12, *Belinda Vos*	Ukiah, CA Sep 28	18	346.0 SSW	*J and S. Tendick*
**B5244**	M	Ashland, OR	Sep 11, *Steve Johnson*	NB CA **(OS)** Oct 3	82	585.8 S	*John Dayton*
**B5893**	M	Ashland, OR	Sep 5, *Lauren Hisatomi*	NB CA **(OS)** Oct 9	79	574.5 S	*John Dayton*
**B5593**	F	Elkton, OR	Sep 3, *B. Slott/K. Hendricks*	NB CA **(OS)** Oct 9	139	754.8 SSE	*John Dayton*
**B5307**	M	Brookings, OR	Aug 31, *Holly Beyer*	LF CA **(OS)** Oct 9	161	595.5 SSE	*John Dayton*
**B5260**	F	Eugene, OR	Jul 30, *Jessica Jankowski*	LF CA **(OS)** Oct 9	65	793.4 S	*John Dayton*
**B3720**	M	Medford, OR	Aug 26, *R and S Coffan*	LF CA **(OS)** Oct 9	55	598.7 S	*John Dayton*
**C0745**	M	Yakima, WA	Aug 31, *David James*	LF CA **(OS)** Oct 9	169	1084.7 S	*John Dayton*
**C3550**	M	Redmond, WA	Sep 3, *Connie Granberg*	Avila Beach, CA Oct 26	136	1392.1 S	*Lyle Rains*
**B2462**	M	Redmond, WA	Aug 31, *Connie Granberg*	Kale/Elm, Bolinas, CA **(OS)** Oct 26	86	1086.3 S	*Paul Cherubini*
**C4407**	M	Ashland, OR	Sep 8, *Becky Spangler*	ML CA **(OS)** Oct 19	173	585.8 S	*John Dayton*
**B3859**	M	Brookings, OR	Sep 6, *Dennis Triglia*	LF CA **(OS)** Oct 20	164	618.0 SSE	*John Dayton*
**C4780**	F	Redmond, WA	Sep 6, *Connie Granberg*	LF CA **(OS)** Oct 20	143	1192.5 S	*John Dayton*
**B5239**	M	Ashland, OR	Sep 4, *Steve Johnson*	NB CA **(OS)** Oct 21	156	585.8 S	*John Dayton*
**B6698**	M	Klamath Falls, OR	Sep 22, *A. King/A. Stercho*	NB CA **(OS)** Oct 21	68	579.4 S	*John Dayton*
**C5043**	M	Elkton, OR	Sep 16, *B. Slott/K. Hendricks*	NB CA **(OS)** Oct 21	68	754.8 SSE	*John Dayton*
**C4114**	F	Gold Hill, OR	Sep 16, *Lee Finney*	NB CA **(OS)** Oct 29	139	616.4 S	*John Dayton*
**C0190**	M	Yakima, WA	Aug 22, *David James*	ML CA **(OS)** Oct 29	110	1083.1 SSW	*John Dayton*
**B6578**	M	Salem, OR	Aug 18, *S. Hazen*	NB CA **(OS)** Oct 30	209	883.5 S	*John Dayton*
**C0677**	M	Yakima, WA	Aug 30, *David James*	NB CA **(OS)** Oct 30	61	1084.7 SSW	*John Dayton*
**C5023**	M	Elkton, OR	Sep 16, *B. Slott/K. Hendricks*	ML CA **(OS)** Nov 1	187	751.6 S	*John Dayton*
**B6806**	F	Eagle Point, OR	Sep 26, *Leigh Leaming*	ML CA **(OS)** Nov 1	117	616.4 S	*John Dayton*
**C4041**	F	Talent, OR	Aug 31, *Belinda Vos*	Ellwood West, Goleta, CA **(OS)** Nov 8	69	904.5 SSE	*Dan Meade*
**B5981**	M	Medford, OR	Sep 20, *R. and S. Coffan*	Atascadero Ck. Goleta, CA **(OS)** Nov 15	56	914.1 SSE	*Charis van der Heide*
**C0694**	M	Yakima, WA	Aug 30, *David James*	ML CA **(OS)** Nov 10	170	1083.1 SSW	*John Dayton*
**B3745**	M	Brookings, OR	Aug 28, *Dennis Triglia*	NB CA **(OS)** Nov 15	80	597.1 SSE	*John Dayton*
**C3451**	M	Redmond, WA	Sep 3, *Connie Granberg*	LF CA **(OS)** Nov 15	183	1190.9 S	*John Dayton*
**C3502**	M	Redmond, WA	Sep 3, *Connie Granberg*	Prefumo Ck., San Luis Obispo, CA **(OS)** Nov 17	75	1380.8 S	*Jessica Griffiths*
**C1475**	M	Yakima, WA	Sep 15, *David James*	LF CA **(OS)** Nov 17	93	1084.7 SSW	*John Dayton*
**C1775**	M	Yakima, WA	Sep 22, *David James*	Kale/Elm, Bolinas, CA **(OS)** Nov 18	57	983.3 SSW	*J. Garcia/M. Monroe*
**C5024**	M	Elkton, OR	Sep 16, *B. Slott/K. Hendricks*	Pismo Beach, CA **(OS)** Nov 19	130	978.5 SSE	*David James*
**C4109**	M	Gold Hill, OR	Sep 14, *Lee Finney*	ML CA **(OS)** Nov 22	118	614.8 SSE	*D. James/J. Dayton*
**B5565**	M	Elkton, OR	Sep 6 *B. Slott/K. Hendricks*	ML CA **(OS)** Nov 22	94	753.2 SSE	*D. James/J. Dayton*
**C4094**	M	Brookings, OR	Oct 6, *Holly Beyer*	NB CA **(OS)** Nov 23	48	606.7 SSE	*D. James/J. Dayton*
**C4399**	F	Talent, OR	Sep 17, *Belinda Vos*	Kale/Elm, Bolinas, CA **(OS)** Nov 24	68	482.8 S	*David James*
**B5443**	F	Elkton, OR	Aug 29, *B. Slott/K. Hendricks*	Alder Ck., Bolinas, OR **(OS)** Nov 24	87	640.5 SSE	*David James*
**B5072**	F	Brookings, OR	Sep 19, *Patsy Haggerty*	Muir Beach, CA, **(OS)** Nov 24	66	487.6 SSE	*Mia Monroe*
**C5015**	F	Elkton, OR	Sep 16, *B. Slott/K. Hendricks*	Encinal Beach, Alameda, CA Nov 29	74	661.4 SSE	*Susan Ramos*
**C3592 (wing)**	M	Redmond, WA	Sep 4, *Connie Granberg*	Redwoods SP, Orick, CA Nov 12	69	714.5 SSW	*Tyler Steeves*
**B5436**	M	Elkton, OR	Aug 27, *B. Slott/K. Hendricks*	ML CA **(OS)** Nov 28	200	754.8 S	*John Dayton*
**C4199**	M	Talent, OR	Sep 5, *Belinda Vos*	Ardenwood Farm, Fremont, CA **(OS)** Dec 7	93	526.2 S	*Daniel La Flash*
**B5754**	M	Talent, OR	Sep 11, *Aleece Townsend*	ML CA **(OS)** Nov 28	121	592.2 S	*John Dayton*
**C4214**	M	Talent, OR	Sep 16, *Belinda Vos*	Pismo Beach, CA **(OS)** Dec 14	114	814.3 SSE	*Darya Veach*
**B5479**	F	Elkton, OR	Sep 1, *B. Slott/K. Hendricks*	LF CA **(OS)** Dec 12	102	754.8 SSE	*John Dayton*
**B6751**	M	Klamath Falls, OR	Sep 20, *Akimi King*	LF CA **(OS)** Dec 15	155	579.4 S	*John Dayton*
**C5078**	M	Elkton, OR	Sep 26, *B. Slott/K. Hendricks*	Pismo Beach, CA **(OS)** Dec 30	107	978.5 SSE	*Betty Sleath/Jere Schafer*
**B5983**	M	Medford, OR	Sep 22, *R. and S. Coffan*	LF CA **(OS)** Jan 7	110	597.1 SSE	*John Dayton*
**C5068**	F	Elkton, OR	Sep 26, *B. Slott/K. Hendricks*	Alder Ck., Bolinas, CA **(OS)** Jan 12	108	642.1 SSE	*David James*
**B2363**	M	Klamath Falls, OR	Sep 12, *A. King/T. Kepple*	Alder Ck., Bolinas, CA **(OS)** Jan 12	122	482.8 SSW	*David James*
**C0065**	M	Yakima, WA	Aug 21, *David James*	Alder Ck., Bolinas, CA **(OS)** Jan 12	144	988.1 SSW	*David James*
**C4195**	M	Talent, OR	Sep 4, *Belinda Vos*	Alder Ck., Bolinas, CA **(OS)** Jan 12	130	482.8 S	*David James*
**B5317**	M	Gold Beach, OR	Oct 6, *Holly Beyer*	Alder Ck., Bolinas, CA **(OS)** Jan 12	98	521.4 SSE	*David James*
**B5466**	M	Elkton, OR	Sep 1, *B. Slott/K. Hendricks*	Los Osos, CA Jan 12	133	954.3 SSE	*Joe Billings*
**B6756**	F	Klamath Falls, OR	Sep 25, *Akimi King*	Pismo Beach, CA **(OS)** Jan 16	113	785.3 SSE	*Peggy Burhenn*
**C1396**	F	Yakima, WA	Sep 13, *David James*	LF CA **(OS)** Jan 18	127	1084.7 SSW	*John Dayton*
**B5321**	M	Brookings, OR	Oct 7, *Holly Beyer*	LF CA **(OS)** Jan 18	103	595.5 SSE	*John Dayton*
**C4091**	F	Gold Beach, OR	Oct 6, *Holly Beyer*	LF CA **(OS)** Jan 18	104	638.9 SSE	*John Dayton*
**B1861**	F	Boise, ID	Sep 4, *Melinda Lowe*	Goleta, CA Mar 2	179	1065.4 SSW	*John McBirney*
**MEAN** **(±SE)**					**89.4 ± 6.7** **(n = 74)**	**640.8 ± 41.5** **(n = 74)**	

**Table 3:** Release, recovery, distance, direction and lifespan data for monarchs captive-reared, tagged and released in the PNW during 2018. **OS** = overwintering site. Santa Cruz overwintering sites: LF = Lighthouse Field State Beach, NB = Natural Bridges State Beach, ML = Moran Lake.

Tag #	Sex	Release Location	Date and Tagger	Recovery Location & Date	Length of Life (Days)	Distance (km) and Direction	Finder
**E5194**	F	Ashland, OR	Aug 24, *Steve Johnson*	Santa Rosa, CA Sep 9	14	416.8 S	*Brenda Tharp*
**C6629**	M	Post Falls, ID	Sep 6, *Bill Ament*	Palouse, WA Sep 16	10	96.6 S	*John Bafenkamp*
**E5146**	F	Medford, OR	Sep 5, *Lynn Kunstman*	Petaluma, CA Sep 18	13	457.1 S	*Gary Danskin*
**E0195**	M	Yakima, WA	Sep 4, *Kristin Gumz*	Glenns Ferry, ID Sep 18	14	360 SE	*Sheila Kramer*
**E3031**	M	Redmond, WA	Sep 24, *Catherine Burns*	Kennydale, WA Sep 29	5	20.9 SSW	*Julie Brown*
**E5798**	M	Elkton, OR	Sep 19, *B. Slott/K. Hendricks*	NB CA **(OS)** Oct 10	49	754.8 S	*Lee Jaffe*
**E5068**	M	Talent, OR	Aug 1, *Belinda Vos*	Hyampom Airport, CA Aug 15	14	188.3 SSW	*Joe Shatos*
**E5844**	M	Elkton, OR	Sep 27, *B. Slott/K. Hendricks*	LF CA **(OS)** Nov 7	114	754.8 S	*John Dayton*
**E5711**	M	Ashland, OR	Sep 14, *Steve Johnson*	Stinson Beach, CA **(OS)** Oct 23	39	478 S	*Judy and Len Chapman*
**E5363**	M	Talent, OR	Aug 17, *Belinda Vos*	ML CA **(OS)** Nov 19	108	590.6 S	*John Dayton*
**E5192**	M	Ashland, OR	Aug 24, *Steve Johnson*	ML CA **(OS)** Nov 26	101	584.2 S	*John Dayton*
**E5125**	F	Applegate, OR	Sep 7, *Linda Kappen*	ML CA **(OS)** Dec 3	114	597.1 S	*John Dayton*
**E1247**	M	Yakima, WA	Sep 26, *David James*	NB CA **(OS)** Nov 22	90	1084.7 SSW	*Jenna Harrison*
**E5524**	M	Klamath Falls, OR	Sep 2, *Akimi King*	Carpinteria, CA **(OS)** Dec 24	113	891.6 SSE	*Joe Billings*
**E5195**	M	Ashland, OR	Aug 27, *Steve Johnson*	Morro Bay Main St, CA **(OS)** Dec 27	122	772.5 SSE	*Joe Billings*
**E5834**	F	Elkton, OR	Sep 22, *B. Slott/K. Hendricks*	Morro Bay Golf C., CA **(OS)** Dec 29	98	949.5 SSE	*Joe Billings*
**B6628**	F	Tulelake, CA	Sep 29, *La Del Bonham*	Davenport, CA Feb 12	136	553.6 SSW	*Joe Paquin*
**E5182**	M	Ashland, OR	Aug 18, *Steve Johnson*	Cayucos, CA Jun 26	312	764.4 SSE	*Jeffrey Germond*
**MEAN** **(±SE)**					**81.3 ± 17.6** **(n = 18)**	**573 ± 68.1** **(n = 18)**	

**Table 4:** Release, recovery, distance, direction and lifespan data for Monarchs captive-reared, tagged and released in the PNW during 2019. **OS** = overwintering site. Santa Cruz overwintering sites: LF = Lighthouse Field State Beach, NB = Natural Bridges State Beach, ML = Moran Lake.

Tag #	Sex	Release Location	Date and Tagger	Recovery Location & Date	Length of Life (Days)	Distance (km) and Direction	Finder
**G1043**	M	Talent, OR	Aug 30, *Belinda Vos*	Arcata, CA Sep 11	12	186.7 SW	*Melissa Mendez*
**G6159**	F	Yakima, WA	Sep 21, *Tim Franks*	Pendleton, OR Sep 29	8	173.8 SE	*Robin Buckle*
**G0533**	F	Brookings, OR	Sep 7, *Dennis Triglia*	Oakland, CA Sep 25	18	506.9 SSE	*Michelle Schubnel*
**G0531**	M	Brookings, OR	Sep 7, *Dennis Triglia*	NB CA **(OS)** Oct 6	68	598.7 SSE	*Chung-Cheng Piao*
**G2506**	M	Eagle Point, OR	Sep 23, *Heather Mauck*	Boulder Ck., CA Oct 13	20	597.1 SSE	*Adrienne Gaughan*
**G2404**	F	Redding, CA	Oct 13, *Harvey/Reynolds*	San Rafael, CA Oct 22	9	288.0 SSW	*Kambia Moezzi*
**G1120**	F	Talent, OR	Sep 14, *Belinda Vos*	Palo Alto, CA Oct 24	40	537.5 SSE	*Karen Krimmer*
**G2006**	M	Elkton, OR	Sep 27, *B. Slott/K. Hendricks*	Alameda, CA Oct 25	28	663.0 SSE	*Gary Keep*
**G0679**	M	Talent, OR	Aug 29, *Belinda Vos*	Woodland, CA Sept 15	17	405.6 SSE	*Rose Cassidy*
**G1769**	M	Ashland, OR	Oct 3, *Paula Caplinger*	NB CA **(OS)** Oct 27	30	584.2 SSE	*Robert Cala*
**G1370**	M	Crescent City, CA	Sep 9, *Vicki Mion*	Ft. Mason, San Fran, CA Nov 3	55	465.1 SSE	*Sandi Wong*
**G1946**	F	Brookings, OR	Sep 21, *Dennis Triglia*	NB CA **(OS)** Nov 3	110	598.7 SSE	*Linda Milom*
**G1615**	M	Bend, OR	Sep 14, *Egertson/Steele*	LF CA **(OS)** Oct 29	61	793.4 S	*John Dayton*
**G1762**	M	Ashland, OR	Oct 3, *Paula Caplinger*	LF CA **(OS)** Nov 26	123	584.2 SSE	*John Dayton*
**G0116**	F	Elkton, OR	Sep 19, *B. Slott/K. Hendricks*	NB CA **(OS)** Oct 29	40	754.8 SSE	*John Dayton*
**G0571**	M	Brookings, OR	Sep 16, *Dennis Triglia*	NB CA **(OS)** Nov 2	37	598.7 SSE	*John Dayton*
**G0785**	M	Brookings, OR	Sep 13, *Holly Beyer*	LF CA **(OS)** Nov 6	151	601.9 SSE	*John Dayton*
**G0752**	F	Brookings, OR	Aug 22, *Holly Beyer*	NB CA **(OS)** Nov 7	47	598.7 SSE	*John Dayton*
**G1772**	M	Ashland, OR	Oct 4, *Paula Caplinger*	NB CA **(OS)** Nov 7	126	584.2 SSE	*John Dayton*
**G0164**	M	Brookings, OR	Aug 11, *Holly Beyer*	Elm/Kale Bolinas, CA **(OS)** Nov 18	99	481.2 SSE	*Paul Cherubini*
**E1478**	F	Brookings, OR	Oct 14, *Holly Beyer*	Pismo SB, CA **(OS)** Nov 20	37	832.0 SSE	*Craig Corwin*
**G0112**	M	Elkton, OR	Sep 19, *B. Slott/K. Hendricks*	Pismo SB, CA **(OS)** Nov 20	62	980.1 S	*Amber Clark*
**G2498**	M	Eagle Point, OR	Sep 21, *Heather Mauck*	Deer Flat, Big Sur, CA Nov 9	49	750.0 S	*Dan Richards*
**G2739**	M	Brookings, OR	Oct 10, *Teresa Lawson*	LF CA **(OS)** Nov 18	111	601.9 SSE	*John Dayton*
**G0556**	M	Brookings, OR	Sep 13, *Dennis Triglia*	Elm/Kale Bolinas, CA **(OS)** Nov 22	70	481.2 SSE	*Juan Garcia*
**G0645**	M	Talent, OR	Aug 28, *Belinda Vos*	ML CA **(OS)** Nov 23	57	592.2 SSE	*John Dayton*
**G1109**	F	Talent, OR	Sep 14, *Belinda Vos*	LF CA **(OS)** Dec 10	113	592.2 SSE	*John Dayton*
**G0927**	M	Brookings, OR	Sep 30, *Holly Beyer*	Presidio, S. Fran. CA Dec 16	77	498.9 SSE	*Jean Koch*
**G1039**	M	Talent, OR	Aug 29, *Belinda Vos*	Huntington, Beach, CA Jan 7	131	1042.9 SSE	*Dan Leichuk*
**E1320**	M	Brookings, OR	Oct 7, *Patsy Haggerty*	Santa Cruz, CA Feb 3	119	608.3 SSE	*Toni Corrigan*
**MEAN** **(±SE)**					**64.2 ± 7.7** **(n = 30)**	**586.1 ± 34.5** **(n = 30)**	

**Table 5:** Re-sighting data for captive-reared tagged monarchs in California in 2017. All data from overwintering sites except for C4378 re-sighted during migration. Santa Cruz overwintering sites: NB = Natural Bridges State Beach, LF = Lighthouse Field State Beach, ML = Moran Lake. Residency = period spent at overwintering site (s).

Tag #	Sex	Origin & Date	1st Resight	2nd	3rd	4th	5th	6th	7th	8th	9th	Residency (Days)
**C4378**	F	Talent, OR Sep 12	Ukiah Sep 28	Ukiah Oct 1								
**B5244**	M	Ashland, OR Sep 11	NB Oct 3	NB Oct 3	NB Oct 6	NB Oct 14	NB Nov 2					29
**B5893**	M	Ashland, OR Sep 5	NB Oct 9	LF Nov 23								45
**B5593**	F	Elkton, OR Sep 9	NB Oct 9	ML Nov 28	LF Jan 10	LF Jan 24	LF Jan 26					108
**B5307**	M	Brookings, OR Aug 31	LF Oct 9	LF Jan 10	LF Jan 26	LF Feb 1	LF Feb 8					122
**B5260**	F	Eugene, OR Jul 30	LF Oct 9	LF Oct 30	LF Nov 2							23
**B3720**	M	Medford, OR Aug 26	LF Oct 9	LF Oct 20								11
**C0745**	M	Yakima, WA Aug 31	LF Oct 9	LF Nov 2	LF Nov 7	LF Dec 12	LF Dec 15	LF Dec 27	LF Feb 16			130
**B5239**	M	Ashland, OR Sep 4	NB Oct 21	LF Jan 13	LF Feb 1	LF Feb 8						110
**C3550**	M	Redmond, WA Sep 3	Avila Beach Oct 26	Morro Bay CG Jan 7								
**B2462**	M	Redmond, WA Aug 31	Elm/Kal Bolinas Oct 26	Alder Bolinas Nov 25								30
**C4407**	M	Ashland, OR Sep 8	ML Oct 19	ML Nov 28	ML Jan 6	ML Jan 10	ML Feb 28					131
**B3859**	M	Brookings, OR Sep 6	LF Oct 20	LH Dec 12	LF Feb 27							131
**C4780**	F	Redmond, WA Sep 6	LF Oct 20	ML Nov 23	ML Dec 16	ML Jan 27						99
**B6698**	M	Klamath Falls, OR Sep 22	NB Oct 21	LF Nov 23	LF Nov 29							39
**C4114**	F	Gold Hill, OR Sep 16	ML Oct 29	ML Nov 15	ML Dec 10	ML Dec 28	ML Jan 15	ML Feb 2				96
**C0190**	M	Yakima, WA Aug 22	ML Oct 29	ML Nov 10	ML Nov 15	ML Nov 23	ML Dec 10					42
**B6578**	M	Salem, OR Aug 18	NB Oct 30	LF Nov 29	ML Dec 17	ML Jan 15	ML Jan 21	ML Jan 24	LF Mar 10			131
**C5023**	M	Elkton, OR Sep 16	ML Nov 1	LF Nov 23	LF Dec 27	LF Dec 30	LF Jan 7	LF Feb 1	LF Feb 16	LF Mar 19		139
**B6806**	F	Eagle Point, OR Aug 26	ML Nov 1	ML Nov 10	ML Nov 22	ML Dec 13	ML Dec 21					51
**C0694**	M	Yakima, WA Aug 30	ML Nov 10	ML Nov 15	LF Feb 5	LF Feb 8	LF Feb 16					98
**C3451**	M	Redmond, WA Sep 3	LF Nov 14	LF Nov 23	LF Nov 29	LF Dec 15	LF Dec 30	LF Jan 10	LF Jan 26	LF Feb 8	LF Feb 23	101
**C1475**	M	Yakima, WA Sep 15	LF Nov 17	ML Dec 17								30
**C5024**	M	Elkton, OR Sep 16	Pismo Nov 19	Pismo Dec 29	Pismo Dec 31	Pismo Jan 24						66
**C4109**	M	Gold Hill, OR Sep 14	ML Nov 22	ML Jan 10								49
**B5565**	M	Elkton, OR Sep 6	ML Nov 22	ML Nov 28	ML Dec 19							27
**B5436**	M	Elkton, OR Aug 27	ML Nov 28	ML Dec 21	ML Dec 23	ML Dec 28	LF Jan 20	LF Mar 15				107
**B5754**	M	Talent, OR Sep 11	ML Nov 28	ML Dec 10	ML Dec 13	ML Jan 10						43
**C4214**	M	Talent, OR Sep 16	Pismo Dec 14	Pismo Jan 8								25
**B6751**	M	Klamath Falls, OR Sep 20	LF Dec 15	LF Dec 22	LF Dec 27	LF Jan 20	LF Jan 26	LF Feb 16				63
**B5983**	M	Medford, OR Sep 22	LF Jan 7	LF Jan 10								3
**B5078**	M	Elkton, OR Sep 26	Pismo Dec 30	Pismo Jan 11								12
**B5321**	M	Brookings, OR Oct 7	LF Jan 18	LF Feb 8	LF Feb 13							26
**C5043**	M	Elkton, OR Sep 16	NB Oct 21	NB Nov 17	LF Nov 23							33
**MEAN** **(±SE)**												**67.2 ± 7.7** **(n = 32)**

**Table 6:** Re-sighting data for tagged captive-reared monarchs in California in 2018. All data from overwintering sites in Santa Cruz: NB = Natural Bridges State Beach, LF = Lighthouse Field State Beach, ML = Moran Lake. Residency = period spent at overwintering site (s).

Tag #	Sex	Origin & Date	1st Resight	2nd	3rd	4th	5th	Residency (Days)
**E5798**	M	Elkton, OR Sep 19	NB Oct 10	NB Nov 7				28
**E5844**	M	Elkton, OR Sep 27	LF Nov 7	LF Nov 19	LF Nov 27	LF Dec 16	ML Jan 19	73
**E5363**	M	Talent, OR Aug 17	ML Nov 19	ML Nov 26	ML Dec 3			15
**E5192**	M	Ashland, OR Aug 24	ML Nov 26	ML Nov 30	ML Dec 3			7
**E5125**	F	Applegate, OR Sep 7	ML Dec 3	ML Dec 27				24
**E1247**	M	Yakima, WA Sep 28	NB Nov 22	NB Dec 27				35
**MEAN** **(±SE)**								**30.3 ± 9.4** **(n = 6)**

**Table 7:** Re-sighting data for captive-reared tagged monarchs in California in 2019. All data from overwintering sites in Santa Cruz: NB = Natural Bridges State Beach, LF = Lighthouse Field State Beach, ML = Moran Lake. Residency = period spent at overwintering site (s).

Tag #	Sex	Origin and Date	1st Resight	2nd	3rd	4th	5th	6th	7th	8th	9th	10th	11th	12th	Residency (Days)
**G0531**	M	Brookings, OR Sep 7	NB Oct 6	NB Oct 29	NB Nov 14										39
**G1769**	M	Ashland, OR Oct 3	NB Oct 27	NB Nov 2											6
**G1946**	F	Brookings, OR Sep 21	NB Nov 3	LF Dec 15	LF Jan 9										67
**G1615**	M	Bend, OR Sep 14	LF Oct 29	NB Nov 14											16
**G1762**	M	Ashland, OR Oct 3	LF Oct 29	LF Nov 14	LF Nov 26	LF Nov 29	LF Dec 5	LF Dec 17	LF Dec 23	LF Jan 17	LF Jan 24	LF Jan 29	LF Feb 4		98
**G0785**	M	Brookings, OR Sep 13	LF Nov 6	LF Nov 18	LF Nov 26	LF Nov 28	LF Nov 29	LF Dec 17	LF Jan 2	LF Jan 6	LF Jan 9	LF Jan 17	LF Jan 23	LF Feb 11	97
**G1772**	M	Ashland, OR Oct 4	NB Nov 7	LF Nov 28	LF Dec 10	LF Dec 23	LF Jan 1	LF Jan 23	LF Jan 25	LF Feb 2	LF Feb 7				92
**G2739**	M	Brookings, OR Oct 10	LF Nov 18	LF Nov 21	LF Nov 29	LF Dec 23	LF Dec 29	LF Jan 2	LF Jan 29						72
**G1109**	F	Talent, OR Sep 14	LF Dec 10	LF Dec 17	LF Jan 9										30
**MEAN** **(±SE)**															**57.4 ± 11.9** **(n = 9)**

**Table 8:** Distance and daily rate of travel of captive-reared-tagged fall-migrating monarchs during 2017–2019.

Tag# (Year)	Release–Recovery Locations	Distance Traveled (km)	Release-Recovery (Days)	Rate of Travel (km/Day)
**B5629 (2017)**	Gold Hill, OR–Jacksonville, OR	20.9	1	20.9
**B6917 (2017)**	Rathdrum, ID–Coeur d’Alene, ID	16.1	3	5.4
**C2564 (2017)**	Rathdrum, ID–Spokane, WA	24.1	4	6.0
**B6839 (2017)**	Rathdrum, ID–Spokane, WA	27.4	3	9.1
**C4433 (2017)**	Rathdrum, ID–Spokane, WA	25.7	2	12.8
**B6865 (2017)**	Rathdrum, ID–Coeur d’ Alene, ID	16.1	3	5.4
**C4772 (2017)**	Redmond, WA–Mercer Island, WA	16.1	7	2.3
**B5553 (2017)**	Elkton, OR–Grants Pass, OR	138.4	5	27.7
**B5554 (2017)**	Elkton, OR–Brookings, OR	191	5	38.3
**B5518 (2017)**	Elkton, OR–Jenner, CA	585.8	14	41.8
**B6679 (2017)**	Klamath Falls, OR–Santa Barbara, CA	877.1	19	46.1
**B3729 (2017)**	Brookings, OR–Vacaville, CA	455.4	31	14.7
**C1143 (2017)**	Yakima, WA–Toppenish, WA	24.1	13	1.8
**C4378 (2017)**	Talent, OR–Ukiah, CA	346.0	16	21.6
**C3550 (2017)**	Redmond, WA–Avila Beach, CA	1392.1	53	26.3
**MEAN ± SE (2017)**		**277.1 ± 104.3**	**11.9 ± 3.6**	**18.7 ± 3.8**
**E5194 (2018)**	Ashland, OR–Santa Rosa, CA	416.8	16	26.0
**C6629 (2018)**	Post Falls, ID–Palouse, WA	96.6	10	9.7
**E5146 (2018)**	Medford, OR–Petaluma, CA	457.0	13	35.1
**E0195 (2018)**	Yakima, WA–Glenns Ferry, ID	579.4	14	41.4
**E3031 (2018)**	Redmond, WA–Kennydale, WA	20.9	5	4.2
**E5068 (2018)**	Talent, OR–Hyampom, CA	188.3	15	12.6
**MEAN ± SE (2018)**		**293.2 ± 90.9**	**12.2 ± 1.7**	**21.5 ± 6.1**
**G1043 (2019)**	Talent, OR–Arcata, CA	186.7	12	15.6
**G6159 (2019)**	Yakima, WA–Pendleton, OR	173.8	8	21.7
**G0533 (2019)**	Brookings, OR–Oakland, CA	506.9	18	28.2
**G0531 (2019)**	Brookings, OR–Santa Cruz, CA	598.7	29	20.6
**G2506 (2019)**	Eagle Point, OR–Boulder Ck., CA	597.1	20	29.9
**G2404 (2019)**	Redding, CA–San Rafael, CA	288.0	9	32.0
**G1120 (2019)**	Talent, OR–Palo Alto, CA	537.5	40	13.0
**G2006 (2019)**	Elkton, OR–Alameda, CA	663.0	28	24.0
**G0679 (2019)**	Talent, OR–Woodland, CA	405.6	17	23.9
**G1769 (2019)**	Ashland, OR–Santa Cruz, CA	584.2	24	24.0
**MEAN ± SE (2019)**		**454.2 ± 56.9**	**20.5 ± 3.2**	**23.3 ± 1.9**
**MEAN ± SE (2017–2019)**		**337.3 ± 57.0**	**14.7 ± 2.1**	**20.7 ± 2.2**

**Table 9:** Migration distances and lifespan of captive-reared tagged monarchs released into smoky or non-smoky air at Brookings, Oregon during August–October 2017. All recoveries made at overwintering sites except where noted.

Tag #	Sex	Release Date	AQI (Air Quality Index)	Recovery Date/Location	Post-Release Length of Life (Days)	Migration Distance (km)
**Smoky: unhealthy air (AQI > 100)**						
**B3729**	M	Aug 24	200 (estimate)	Sep 24 Vacaville, CA *(pre overwintering)*	31	455.4
**B3745**	M	Aug 28	198	Nov 15, Natural Bridges, Santa Cruz, CA	80	597.1
**B5307**	M	Aug 31	200 (estimate)	Oct 9, Lighthouse Field, Santa Cruz, CA	161	595.5
**B3859**	M	Sep 6	304	Oct 20, Lighthouse Field, Santa Cruz, CA	164	618.0
**MEAN ± SE**			**225.5 ± 26.2**		**109 ± 32.5**	**566.5 ± 37.4**
**Non-smoky: healthy air (AQI < 100)**						
**B5072**	F	Sep 19	30	Nov 24, Muir Bch, CA	66	487.6
**B5317**	M	Oct 6	<50	Jan 12, Bolinas, CA	98	521.4
**C4091**	F	Oct 6	<50	Jan 18, Lighthouse Field, Santa Cruz, CA	104	638.9
**C4094**	M	Oct 6	<50	Nov 24, Natural Bridges, Santa Cruz, CA	48	606.7
**B5321**	M	Oct 7	<50	Jan 18, Lighthouse Field, Santa Cruz, CA	103	595.5
**MEAN ± SE**			**<46 ± 4.0**		**83.8 ± 11.3**	**570.0 ± 28.2**

**Table 10:** Length of life and migration distances of captive-reared tagged monarchs released into smoky air in the Medford-Ashland region of southern Oregon during August–September 2018. All recoveries made at overwintering sites except where noted.

Tag #	Sex	Release Date/Location	AQI (Air Quality Index)	Recovery Date/Location	Post-Release Longevity (Days)	Migration Distance (km)
**E5068**	M	Aug 1, Talent, OR	100	Aug 15 Hyampom, CA *(pre-wintering)*	14	188.3
**E5363**	M	Aug 17, Talent, OR	142	Nov 19, Moran Lake, S. Cruz, CA	93	590.6
**E5182**	M	Aug 18, Ashland, OR	172	Jun 26, Cayucos, CA *(post-wintering)*	312	764.4
**E5194**	F	Aug 24, Ashland, OR	165	Sep 9, Santa Rosa, CA *(pre-wintering)*	16	416.8
**E5192**	M	Aug 24, Ashland, OR	165	Nov 26, Moran Lake, S. Cruz, CA	94	584.2
**E5195**	M	Aug 27, Ashland, OR	50	Dec 27, Morro Bay, CA	122	772.5
**E5146**	F	Sep 5, Medford, OR	177	Sep 18, Petaluma, CA *(pre-wintering)*	13	457.1
**E5125**	F	Sep 7, Applegate, OR	125	Dec 3, Moran Lake, S. Cruz, CA	94	584.2
**E5711**	M	Sep 14, Ashland, OR	143	Oct 23, Stinson Beach, CA	39	478.0
**Mean (±SE)**			**137.7 ± 13.7**		**88.6 ± 31.2**	**537.3 ± 60.1**

**Table 11:** Length of life and migration distances of captive-reared tagged *Ophryocystis elektroscirrha* (OE)-infected and uninfected monarchs released from Yakima, WA during August–September 2017. All recoveries made at overwintering sites in California.

Tag #	Sex	Release Date	Recovery Date/Location	Post-Release Length of Life (Days)	Migration Distance (km)
**OE-infected**					
**C 0065**	M	Aug 21	Jan 12, Bolinas CA	144	988.1
**C0190**	M	Aug 22	Oct 29, Moran Lake, S. Cruz, CA	110	1083.1
**C0694**	M	Aug 30	Nov 10, Moran Lake, S. Cruz, CA	170	1083.1
**C1396**	F	Sep 13	Jan 18, Lighthouse Field, S. Cruz, CA	127	1084.7
**C1475**	M	Sep 15	Nov 17 Lighthouse Field, S. Cruz, CA	93	1084.7
**C1775**	M	Sep 22	Nov 18, Bolinas, CA	57	983.3
**MEAN (±SE)**				**116.8 ± 16.2**	**1051.2 ± 20.7**
**Non-infected**					
**C0677**	M	Aug 30	Oct 30 N. Bridges, S. Cruz, CA	61	1084.7
**C0745**	M	Aug 31	Oct 9, Lighthouse Field, S. Cruz, CA	169	1084.7
**MEAN (±SE)**				**115.0 ± 54.0**	**1084.7 ± 0.0**

## Data Availability

Not applicable.
